# Screening of serum biomarkers in patients with PCOS through lipid omics and ensemble machine learning

**DOI:** 10.1371/journal.pone.0313494

**Published:** 2025-01-07

**Authors:** Ji-ying Chen, Wu-jie Chen, Zhi-ying Zhu, Shi Xu, Li-lan Huang, Wen-qing Tan, Yong-gang Zhang, Yan-li Zhao

**Affiliations:** 1 Department of Obstetrics and Gynecology, Shenzhen Longhua District Central Hospital, Shenzhen, China; 2 Department of Medical Laboratory, Shenzhen Longhua District Central Hospital, Shenzhen, China; 3 Department of General Practice, Shenzhen Longhua District Central Hospital, Shenzhen, China; 4 Department of Clinical Laboratory, Shenzhen Longhua District Central Hospital, Shenzhen, China; Federal University of Minas Gerais: Universidade Federal de Minas Gerais, BRAZIL

## Abstract

Polycystic ovary syndrome (PCOS) is a primary endocrine disorder affecting premenopausal women involving metabolic dysregulation. We aimed to screen serum biomarkers in PCOS patients using untargeted lipidomics and ensemble machine learning. Serum from PCOS patients and non-PCOS subjects were collected for untargeted lipidomics analysis. Through analyzing the classification of differential lipid metabolites and the association between differential lipid metabolites and clinical indexes, ensemble machine learning, data preprocessing, statistical test pre-screening, ensemble learning method secondary screening, biomarkers verification and evaluation, and diagnostic panel model construction and verification were performed on the data of untargeted lipidomics. Results indicated that different lipid metabolites not only differ between groups but also have close effects on different corresponding clinical indexes. PI (18:0/20:3)-H and PE (18:1p/22:6)-H were identified as candidate biomarkers. Three machine learning models, logistic regression, random forest, and support vector machine, showed that screened biomarkers had better classification ability and effect. In addition, the correlation of candidate biomarkers was low, indicating that the overlap between the selected biomarkers was low, and the combination of panels was more optimized. When the AUC value of the test set of the constructed diagnostic panel model was 0.815, the model’s accuracy in the test set was 0.74, specificity was 0.88, and sensitivity was 0.7. This study demonstrated the applicability and robustness of machine learning algorithms to analyze lipid metabolism data for efficient and reliable biomarker screening. PI (18:0/20:3)-H and PE (18:1p/22:6)-H showed great potential in diagnosing PCOS.

## Introduction

Polycystic ovary syndrome (PCOS) is a multifactorial, complex, and heterogeneous disorder characterized by hyperandrogenism, menstrual irregularities, infertility, and polycystic ovaries on ultrasound [1]. As an endocrine disorder, PCOS affects about 7%-8% of women of reproductive age [2]. Essential factors in its pathogenesis are hyperinsulinemia, insulin resistance, and dyslipidemia [3]. Endocrine and metabolic abnormalities, including infertility, obesity, type 2 diabetes, and increased luteinizing hormone (LH), are common in women with PCOS [4]. In many cases, lifestyle changes [5] and complementary and alternative medicines are the first-line treatments of choice [6]. Despite the high incidence of PCOS, its attendant morbidity is likely to be underdiagnosed. Therefore, the search for molecular markers that can identify and treat PCOS early and understand its pathogenesis is conducive to taking appropriate interventions for early treatment and providing new therapeutic targets.

Biomarkers are crucial in diagnosing and monitoring PCOS [7, 8]. For example, elevation of the anti-Mullerian hormone (AMH) level is associated with the diagnosis of PCOS, which can be used as an indicator to assess ovarian reserve function [9]. A decrease in lipocalin levels has been associated with insulin resistance and abnormal lipid metabolism in patients with PCOS [10]. Abnormal lipid metabolism is also common in patients with PCOS [11]. Studies have shown specific changes in the lipid profile of PCOS patients, such as abnormal phosphatidylcholine (PC) metabolism, decreased polyunsaturated fatty acids (PUFAs), and increased long-chain saturated fatty acids (LSFAs) [12, 13]. Therefore, identifying non-invasive PCOS biomarkers has become a priority in PCOS research.

Metabolomics looks for relative relationships between specific metabolites, diseases, and their phenotypic changes through qualitative and quantitative analysis of blood, urine, feces, and other body fluids [14]. It is a powerful exploratory tool for discovering new diagnostic molecules or biomarkers, providing evidence for diagnosing and treating diseases, including PCOS [15]. The rapid development of metabolomics has led to renewed interest in metabolism and the role of small molecule metabolites in many biological processes [16]. Untargeted metabolomics is a “hypothesis-generating discovery strategy” that compares sample groups (e.g., cases versus controls), identifies metabolome, and establishes (early indications) perturbations [17, 18]. Therefore, this study further screened the lipid metabolites in the serum of PCOS patients using untargeted lipidomics.

Machine learning is an emerging field of medicine, and numerous resources are devoted to the fusion of computer science and statistics to medical problems [19]. It is a branch of computer science and statistics that generates predictive or descriptive models by learning from training data rather than through rigorous programming [20]. Computational modeling and simulations can help interpret and understand critical biomarkers of physiological significance extracted from machine learning [21]. In the study of PCOS, Silva IS et al. used a machine learning model to classify clinical and laboratory variables associated with novel phenotypes of PCOS into phenotypically distinct clusters, helping to guide more personalized and effective treatments for PCOS [22]. Zhang X et al. used Raman spectroscopy combined with a machine learning algorithm to analyze and characterize metabolic changes in follicular fluid and plasma samples from PCOS patients [23]. These studies demonstrate the critical role of machine learning in diagnosing PCOS markers.

Based on the above background, this study is the first to combine untargeted lipidomics with machine learning as a powerful strategy for efficient and accurate screening of POCS biomarkers. In this study, we screened the key lipid metabolites in the serum of PCOS patients. We constructed a more efficient diagnostic marker panel model based on the feature selection technology of ensemble learning for clinical application.

## Materials and methods

### Clinical samples

152 PCOS patients (PCOS, EG) and 50 non-PCOS subjects (Control, CG) from Shenzhen Longhua District Central Hospital participated. Table 1 summarises the clinical characteristics of PCOS patients and non-PCOS subjects. PCOS was diagnosed by the European Society of Human Reproduction and Embryology/American Society for Reproductive Medicine (ESHRE/ASRM) Rotterdam’s Criteria 2003 [24]. This study collected general information about the study subjects, including gender, age, body mass index, and related hormone levels. Diagnostic criteria included scanty menstruation, amenorrhea, or irregular uterine bleeding; fulfilling one of the two criteria (hyperandrogenic clinical manifestations or hyperandrogenemia; ultrasound showing bilateral or unilateral ovarian polycystic changes and/or increased volume), and excluding other diseases that might cause hyperandrogenism and ovulatory abnormalities. Other inclusion criteria included Han nationality, not taking any hormonal drugs (e.g., estrogen, progesterone, birth control pills, etc.) in the last 6 months. Exclusion criteria included hyperadrenocorticism, tumors secreted by the ovaries or adrenal glands, and pharmacological hyperandrogenism. The study was performed after obtaining verbal informed consent from all participants and was approved by the Shenzhen Longhua District Central Hospital Committee (Approval number: 2022-005-01). Samples were collected from March 1, 2022, until December 31, 2022. All participants provided the written informed consent.

**Table 1:** Characteristics of control and patients with PCOS [average (minimum, maximum)].

Characteristics	Control group (n = 50)	PCOS group (n = 152)	Welch-corrected t	*p*-value
Age (years)	29.2 (21, 36)	26.04 (15, 38)	4.543	<0.0001
Height (cm)	156.8 (145, 168)	156.6 (142, 172)	0.2077	0.8360
Weight (kg)	51.37 (43, 77)	52.62 (40, 90)	1.036	0.3023
Luteinizing hormone (LH)	6.124 (2.79, 26.85)	10.69 (0.18, 33.84)	5.690	<0.0001
Follicle stimulating hormone (FSH)	7.264 (3.94, 13.99)	5.615 (1.7,10.51)	4.804	<0.0001
Progesterone (P)	0.3702 (0.08, 1.57)	0.9541 (0.05, 37.44)	1.674	0.0961
Prolactin (PRL)	19.21 (5.47, 65.12)	17.06 (2.03, 88.25)	1.220	0.2258
Testosterone (T)	0.2707 (0.025, 0.475)	0.4093 (0.023, 0.94)	6.382	<0.0001
Estradiol (E2)	57.41 (25.18, 320.6)	59.09 (11.38, 1036)	0.1849	0.8535

### Untargeted lipidomics analysis

Serum samples were collected from PCOS patients and non-PCOS subjects. The blood samples were stored in anticoagulation tubes, centrifuged at 3000 rpm for 10 min, and then the upper serum was transferred to Eppendorf tubes and stored at -80°C until further processing. Lipids were extracted using the MTBE method. Briefly, samples were homogenized with 200 μL water and 240 μL methanol. Then 800 μL of MTBE was added, and the mixture was ultrasound 20 min at 4°C followed by sitting still for 30 min at room temperature. The solution was centrifuged at 14000 g for 15 min at 10°C, and the upper organic solvent layer was obtained and dried under nitrogen. Reverse phase chromatography was selected for LC separation using the CSH C18 column (1.7 μm, 2.1 mm × 100 mm, Waters). The samples were separated by UHPLC Nexera LC-30A and analyzed by mass spectrometry using a Q-Exactive Plus in positive and negative mode, respectively [25–27]. The data obtained from the positive and negative ion patterns were analyzed using Lipid Search. The lipid class and the number of lipid species identified in each class were identified by positive and negative ion patterns. Changes in lipid content reflect changes in lipid function, and functional discussions and subsequent biofunctional studies were conducted on each of the changed lipid classes.

### Feature weight calculation

Integration was based on the combination of multiple feature selection methods. Substances (such as proteins and metabolites) frequently selected in feature selection methods were rewarded. The reward scores obtained in each feature selection method were combined to calculate each substance’s comprehensive weight value.

### Selection of candidate biomarkers

The distribution of lipid metabolites in different classifications was first demonstrated to select candidate biomarkers efficiently. Orthogonal partial least squares discriminant analysis (OPLS-DA) modelling of lipid metabolites was performed on samples from both groups with 100 simulation replications. Lipid metabolites were compared between the two groups and screened for metabolites with fold change > 2 or fold change < 0.5 and adjusted P value < 0.05. The lipid metabolites in the OPLS-DA model were ranked according to the magnitude of the Variable Importance for the Projection (VIP) value, and the top 20 were selected. The VIP value obtained from the OPLS-DA model can be used to measure the strength of the influence of the expression pattern of each lipid molecule on the categorical discrimination of each group of samples and the ability to explain them and to excavate biologically significant differential lipid molecules. Generally, lipid molecules with VIP > 1 contribute substantially to model interpretation. To avoid overfitting the supervised model during the modelling process, a permutation test was utilized to test the model and ensure its validity. After obtaining the classifications of differential lipid metabolites, we analyzed the associations between the classifications of these differential metabolites and the clinical indexes using the Mantel test. We detected the associations between the classifications of the differential metabolites using Pearson correlation analysis. A receiver operating characteristic (ROC) analysis was utilized to assess the strength of the influence of candidate markers on the model’s AUC value. AUC value was a familiar indicator performed to evaluate the pros and cons of a two-class model. The full name of AUC was the area under the curve, which was the area under the ROC curve. A higher AUC value usually indicates a better classification effect for the model.

### Validation and evaluation of biomarkers

Three commonly used machine learning models were used: logistic regression (LR), random forest (RF), and support vector machine (SVM) to evaluate the effect of candidate biomarkers on the classification model. The candidate markers were validated respectively. LR is particularly suited to binary classification problems. In lipidomic data analysis, constructing a logistic model by LR allows for assessing how individual lipids or lipid combinations affect specific biological phenotypes. It can be used to determine the association between a particular lipid or lipid module and a specific phenotype (e.g., presence or absence of disease). This has important implications for understanding how specific lipids affect disease risk. RF improves prediction accuracy by building multiple decision trees and combining their predictions, and it is ideally suited for dealing with complex datasets with many variables. RF can be used to identify the essential lipids that differentiate between different phenotypes and assess the importance of each lipid in the model, helping us identify the lipids that have the most significant impact on the phenotype. SVM is suitable for complex classification problems because it best distinguishes different classes by separating hyperplanes in multidimensional space. In lipidomic data, SVM can accurately classify samples based on lipid expression levels, such as separating diseased patients from healthy controls. SVM is particularly well suited to datasets with a small number of samples but a large number of features.

ROC curve analysis on the horizontal axis indicated a false positive rate (FPR, 1-specificity), which referred to the percentage of accurate negative samples incorrectly identified as positive. The vertical axis representss the actual positive rate (TPR), also known as sensitivity, which refers to the proportion of actual positive samples correctly judged as positive. The single model index mainly depended on the area value (AUC value) under the ROC curve, specificity, and sensitivity.

The importance coefficients of candidate biomarkers were calculated using the classification model constructed by the RF algorithm, which was used to compare the contribution of each biomarker to the model. The expression levels of candidate biomarkers in each comparison group were calculated. Pearson correlation coefficient was calculated for the expression levels of the candidate biomarkers. Generally speaking, the lower the correlation between the biomarkers in diagnostic panels, the lower the overlap between the selected biomarkers, and the more optimized the panel combination.

### Construction and validation of the diagnostic panel model

The biomarkers diagnostic panel model was constructed using the LR algorithm. LR is a commonly used classification model that can predict the probability of an event and analyze its influencing factors. Biomarkers LR model formula: p = 1/(1+e^-z^). The expression level value of the biomarker was brought into the probability value p calculated by the panel model formula. If the p-value exceeded the cutoff value, it was considered a positive diagnosis. To obtain this cutoff, we used Youden’s index to determine the optimal cutoff value for diagnostic decisions [28]. When the sensitivity and specificity were given the same weight, the cutoff value corresponding to the maximum Youden’s index was the best critical point for biomarker identification ability [29], because the sum of sensitivity and specificity was the maximum at this point. The best cutoff could have good sensitivity and specificity at the same time. Finally, based on the expression of candidate biomarkers in the sample (the original data set was divided into training set and test set, or new validation set data was provided), ROC analysis was performed using the diagnostic model constructed above.

### Statistical analysis

Graphpad Prism8.0 software was used for statistical analysis. If the normal distribution was met, data were expressed as (x±s), and a t-test was used between groups. If it did not conform to a normal distribution, data were expressed as average (minimum, maximum), and the Mann-Whitney U test was used between groups. *P* value < 0.05 indicated that the difference was statistically significant.

## Results

### Characteristics of control and patients with PCOS

First, we assessed the subjects’ clinical information. Table 1 shows the essential clinical information of control (n = 50) and PCOS (n = 152) subjects. Among them, Height, Weight, Body mass index (BMI), Progesterone (P), Prolactin (PRL), and Estradiol (E2) had no significant difference between the control and PCOS groups (*P* value>0.05). While Age, LH, follicle-stimulating hormone (FSH), LH/FSH, and Testosterone (T) were significantly different between the control and PCOS groups (*P* value <0.05).

### Classification of differential lipid metabolites

**Fig 1:**
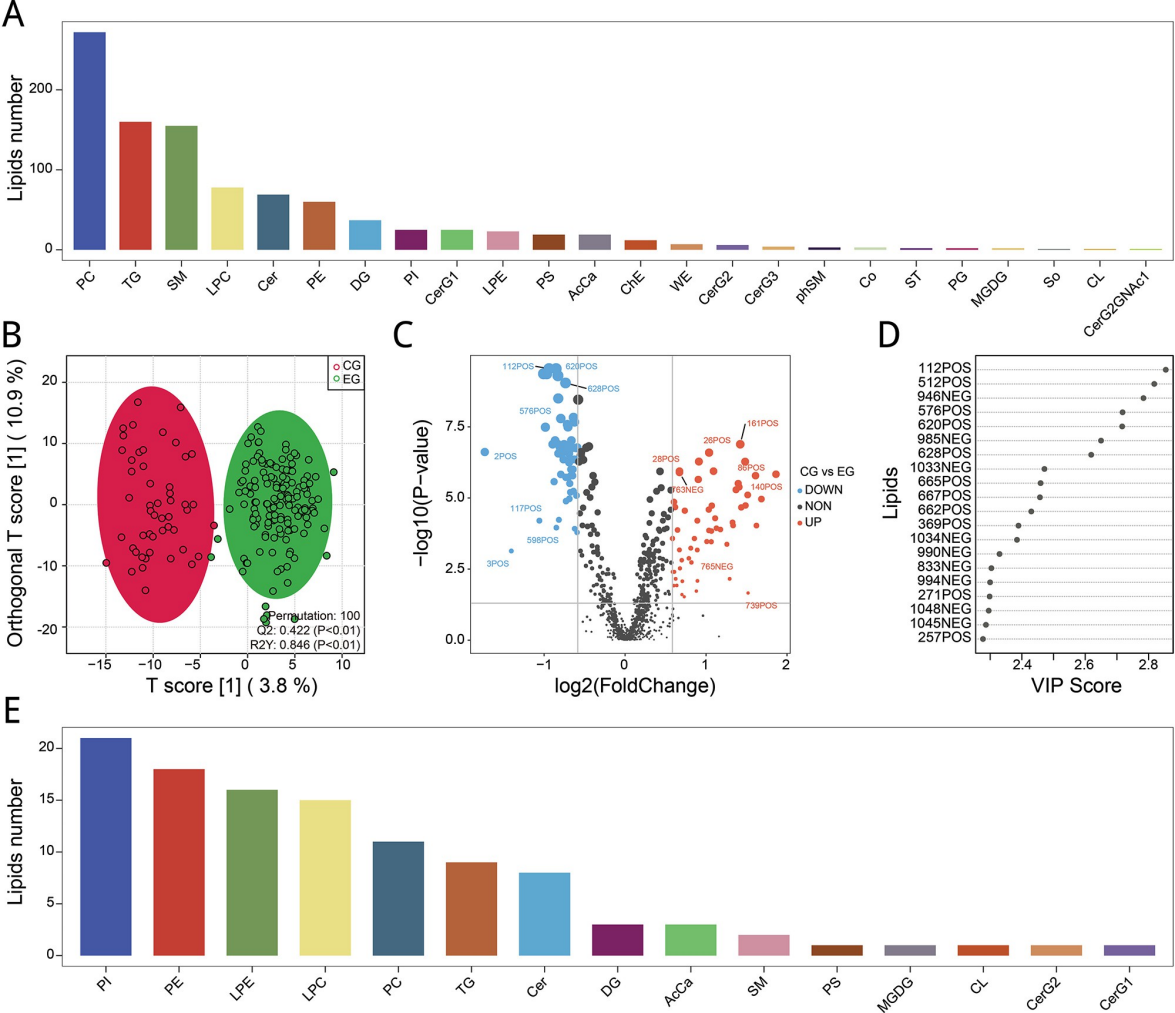
Classification of differential lipid metabolites. (A) Distribution of lipid metabolites in different classifications. Abscissa represents classification, and the ordinate represents a number. (B) OPLS-DA model of Control and PCOS samples. (C) Comparative volcanic map of lipid metabolites between Control and PCOS groups. The red dots represent up-regulated metabolites in the Control group, while the blue dots represent down-regulated metabolites in the Control group. The size of the dots is adjusted to the adjusted P value. The abscissa represents log2 (fold change), and the ordinate represents -log10 (P value). (D) Top 20 metabolites with VIP value in the OPLS-DA model. (E) Classification of differential lipid metabolites.

We collected serum from Control (CG) and PCOS (EG) subjects for untargeted lipidomics analysis to screen candidate biomarkers for PCOS. First, the distribution of lipid metabolites was shown in different classifications (Fig 1A). Then, OPLS-DA modeled the lipid metabolites of the two groups of samples, and 100 simulation repetitions were conducted. As shown in Fig 1B, the model’s predictive ability (Q2 value) was significant, and the explanation ability of sample differentiation between groups was significant and good (R2Y). The lipid metabolites between the two groups were compared, and the metabolites with fold change > 2 or fold change < 0.5 and adjusted P value < 0.05 were screened. The top 8 lipid metabolites of difference included 28POS, 26POS, 161POS, 86POS, 763NEG, 140POS, 765NEG, and 739POS (Fig 1C). Next, the lipid metabolites in the OPLS-DA model were sorted according to the VIP value. The top 20 (112POS, 512POS, 946NEG, 576POS, 620POS, 985NEG, 628POS, 1033NEG, 665POS, 667POS, 662POS, 369POS, 1034NEG, 990NEG, 833NEG, 994NEG, 271POS, 1048NEG, 1045NEG, and 257POS) were selected, suggesting that these metabolites were important for distinguishing Control from PCOS groups (Fig 1D). In addition, the detected differential lipid metabolites were annotated to show the classification of differential lipid metabolites between the Control and PCOS groups (Fig 1E).

### Associations between differential lipid metabolites and clinical indexes

**Fig 2:**
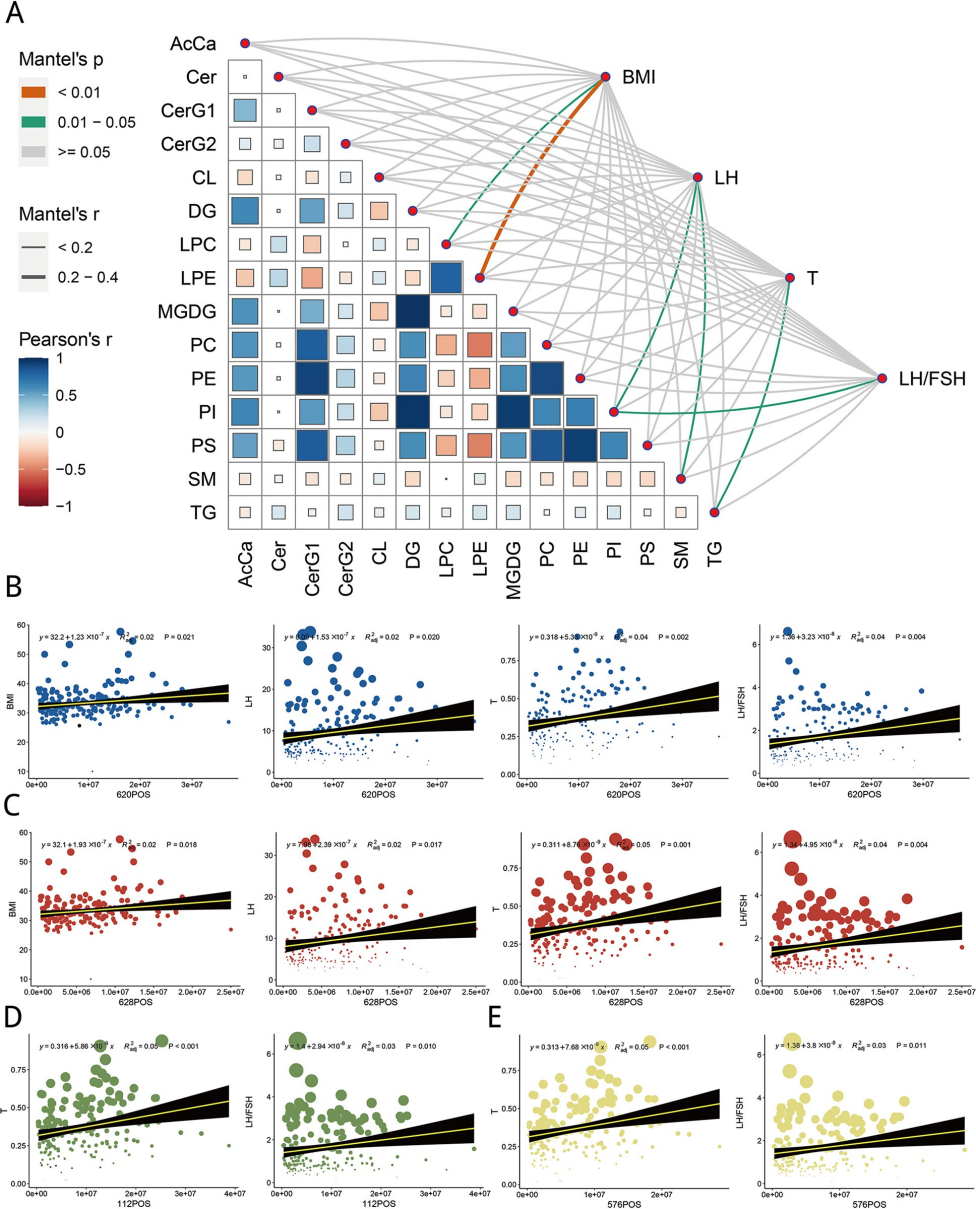
Associations between differential lipid metabolites and clinical indexes. (A) Associations between differential metabolite classification and clinical indexes. B and C. 620POS and 628POS metabolites are significantly associated with clinical indexes (BMI, LH, T, and LH/FSH). D and E. 112POS and 576POS metabolites are associated considerably with clinical indexes (T and LH/FSH).

After obtaining the classification of differential lipid metabolites, we used the Mantel test to analyze the association between the classification of these differential metabolites and clinical indexes and Pearson correlation analysis to detect the association between the classification of differential metabolites. The results showed that LPC and LPE were significantly correlated with BMI, PI and SM were correlated considerably with LH, TG was significantly correlated with testosterone (T), and PI was significantly associated with LH/FSH. Moreover, DG and MGDG classification, PE and PS, PI and DG were significantly positively correlated. There was a significant negative correlation between LPE and PS and between LPE and PC (Fig 2A). Previously, we obtained the central metabolites that differ between the Control and PCOS groups and the top 20 that contribute significantly to the OPLS-DA model (Fig 1C and 1D). Therefore, we examined the relationship between these different lipid metabolites and clinical indexes. We found that 620POS and 628POS were significantly associated with clinical indexes (BMI, LH, T, and LH/FSH), and 112POS and 576POS were significantly associated with clinical indexes (T and LH/FSH, Fig 2C–2E). These results indicate that these metabolites differ between groups and have close effects on corresponding clinical indicators.

### Screening of candidate biomarkers for PCOS

**Fig 3:**
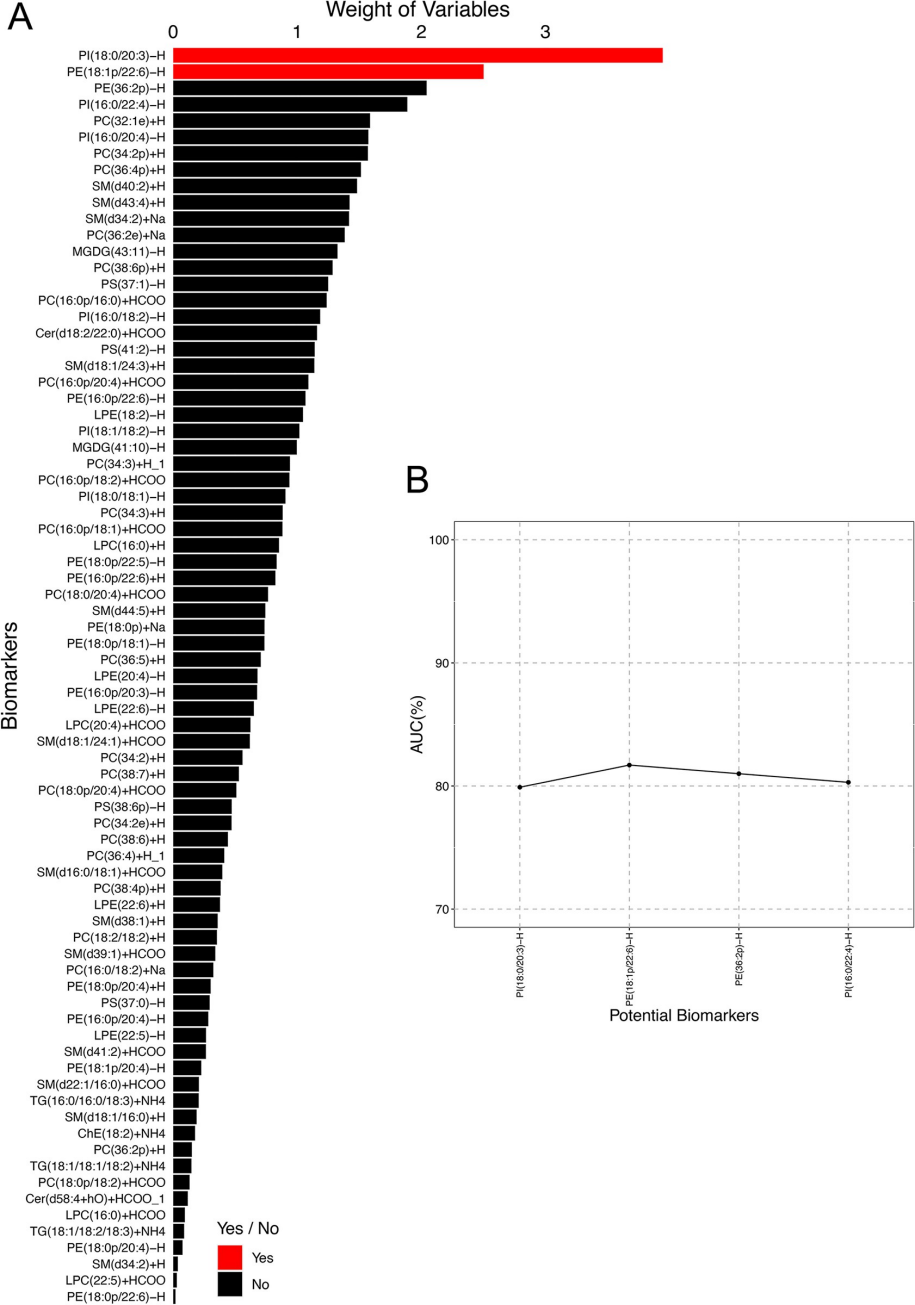
Screening of candidate biomarkers for PCOS. (A) Ranking of candidate biomarkers weight value. Abscissa: weight value; ordinate: substance name; black: biological macromolecules that did not pass the screening criteria; red: candidate biomarkers. (B) AUC cumulative trend plot of candidate biomarkers classification model. Abscissa: candidate biomarkers; ordinate: AUC cumulative value.

Furthermore, Fig 3A shows the ranking of candidate biomarkers by weight value. The larger the weight value, the more significant the contribution of the substance in distinguishing the Control sample from the PCOS sample. Among them, the top 4 were PI (18:0/20:3)-H, PE (18:1p/22:6)-H, PE (36:2p)-H, and PI (16:0 /22:4)-H. To effectively select candidate biomarkers, we used ROC analysis to evaluate the impact strength of each substance on the AUC value of the model. AUC accumulation curve showed that the substances with the top 2 weights notably facilitated sample classification ability; In contrast, substances ranked after 2nd place no longer contribute significantly to classification ability, so PI (18:0/20:3)-H and PE (18:1p/22:6)-H were selected as candidate biomarkers (Fig 3B).

### Validation of biomarkers for PCOS

**Fig 4:**
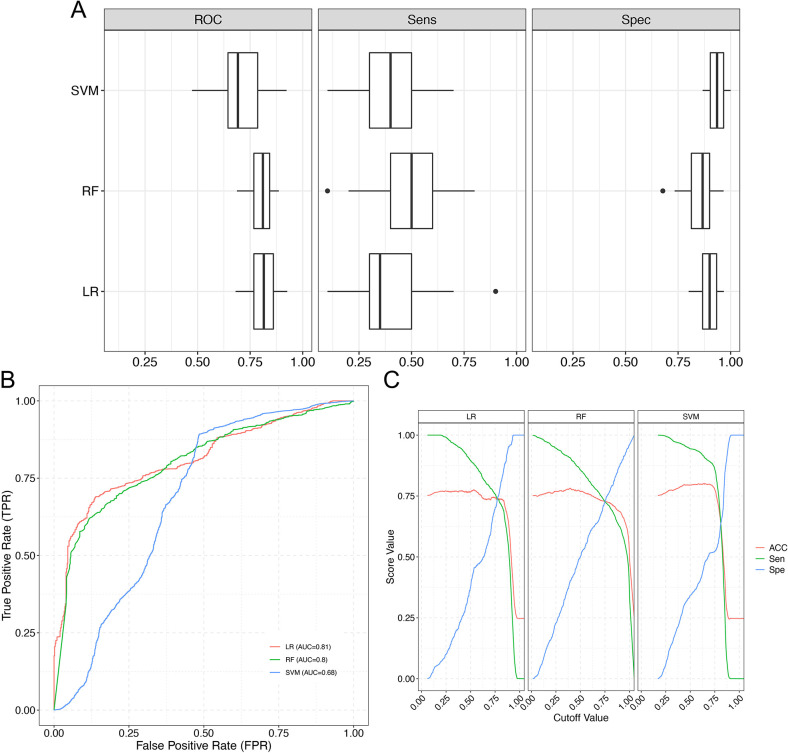
Validation of biomarkers for PCOS. (A) Boxplots of ROC analysis, specificity, and sensitivity for three single model assessments. Abscissa: AUC, specificity and sensitivity scores, the closer it is to 1, the better; ordinate: Three algorithm models used, LR is logistic regression, SVM is support vector machine, and RF is random forest. (B) ROC plots for three single model evaluations. Abscissa: false positive (1-specificity); ordinate: true positive (sensitivity); green curve: random forest ROC curve; blue curve: support vector machine ROC curve; red curve: logistic regression ROC curve; the lower right corner is the AUC value of each model curve. (C) Accuracy, sensitivity, and specificity curves of the three single model evaluations. The three graphs are the accuracy, actual positive rate and accurate negative rate curves of LR, SVM, RF model, abscissa: cutoff value; ordinate: percentage; red curve: accuracy; green curve: sensitivity; blue curve: specificity.

To evaluate the effect of candidate biomarkers on classification models, three commonly used machine learning models, LR, RF, and SVM, were used to verify the above screening results. Fig 4A and 4B showed the three models’ AUC, specificity and sensitivity results in boxplots and ROC curves, respectively. Among them, the closer the area under the ROC curve was to 1, the greater the clinical diagnostic efficacy, and the higher the specificity and sensitivity indicators, the better the efficacy. The results showed that all evaluation indicators had satisfactory results, indicating that the selected subsequent biomarkers had better classification ability and effect. Fig 4C further shows the accuracy, sensitivity, and specificity values of the three ROC models and the curve diagram of the relationship between the cutoff values. The ROC curve comprised multiple sensitivity and false favourable rates (1-specificity). In Fig 4C, each group of accuracy, sensitivity and specificity in the three models has a corresponding cutoff value, and the ROC curve can be used to determine the best cutoff value and its corresponding accuracy, sensitivity and specificity.

### Characterization of biomarkers for PCOS

**Fig 5:**
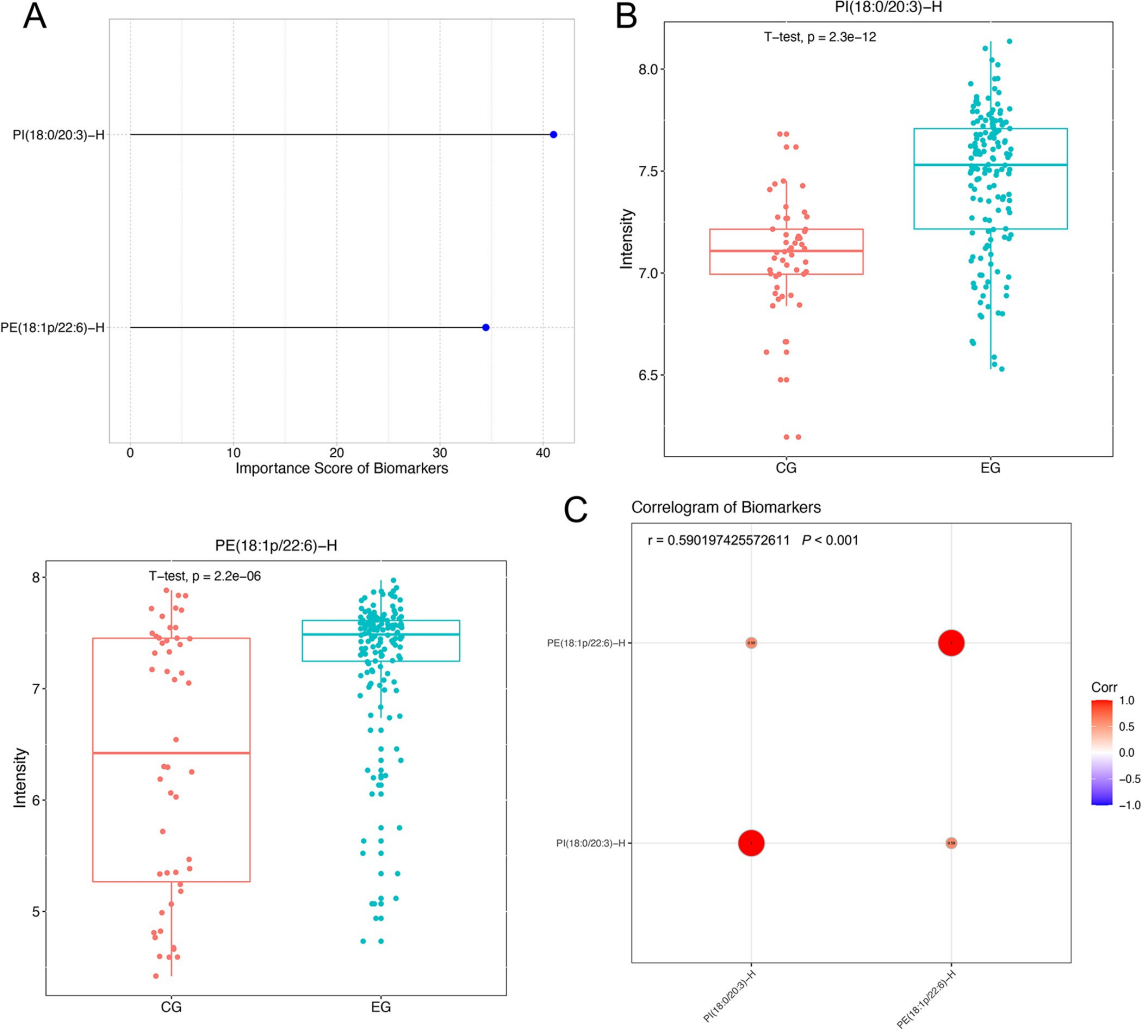
Characterization of biomarkers for PCOS. (A) Random forests calculate the importance of biomarkers. Abscissa: importance; ordinate: substance name. (B) Boxplots of the expression levels of PI (18:0/20:3)-H and PE (18:1p/22:6)-H in each sample. (C) Correlation analysis graph. Abscissa: substance name; ordinate: substance name; circle: relationship coefficient, the larger the correlation, the larger the area and the darker the color; color: red is positive correlation, blue is a negative correlation.

Then, we used the classification model constructed by the above RF algorithm to calculate the importance coefficient of candidate biomarkers and compare the contribution of each biomarker to the model. The higher the importance coefficient of candidate biomarkers, the closer the relationship between the biomarker and the classification group, and the more significant the contribution to distinguishing different groups (Fig 5A). The results of expression level analysis showed that the selected candidate biomarkers PI (18:0/20:3)-H and PE (18:1p/22:6)-H had highly significant differential expression in the comparison group (Fig 5B). Fig 5C shows the correlation between biomarkers in the diagnostic panel. The results showed that candidate biomarkers had low correlation, indicating that the overlap between the selected biomarkers was low, and the combination of panels was more optimized.

### Construction and ability evaluation of PCOS diagnosis panel model

**Fig 6:**
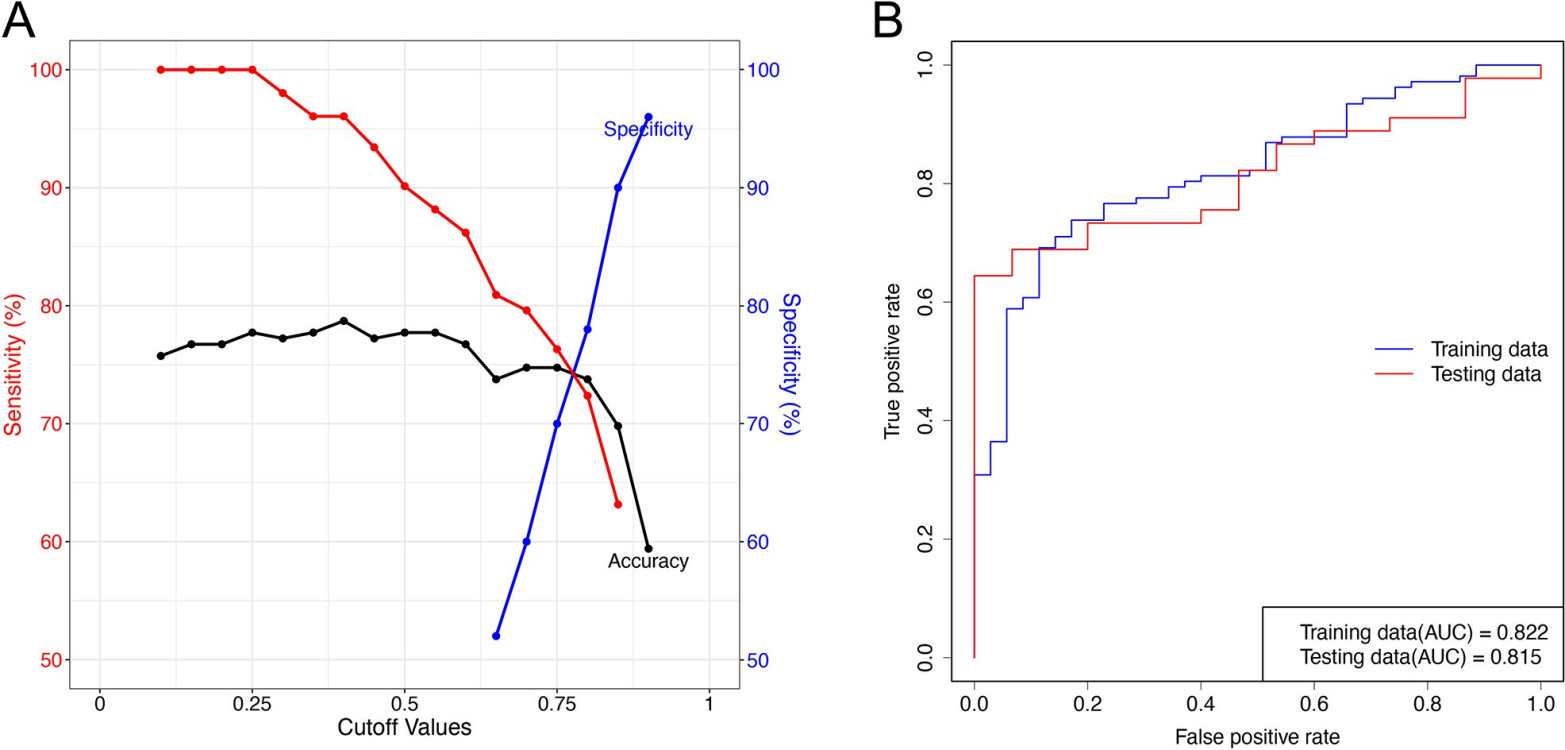
Construction and ability evaluation of PCOS diagnosis panel model. (A) Cutoff trend chart. (B) ROC curve.

The biomarkers diagnostic panel model was constructed using the LR algorithm. Table 2 shows the LR coefficient of biomarkers. The expression levels of the biomarkers were put into the probability p calculated by the panel model formula (biomarkers LR model formula: p = 1/(1+e^-z^), z = -19.62541206+2.249933588*NEG1033+0.634268314*NEG877) if the p-value exceeds the cutoff value, the diagnosis was positive. We used Youden’s index to determine the optimal cut-off value for diagnostic decisions to obtain this cut-off value. Fig 6A shows the variation in the relationship between accuracy and cutoff value, as well as sensitivity and specificity and cutoff value. The cutoff value was 0.83. ROC analysis was performed using the diagnostic model constructed above based on the expression levels of the biomarkers in the samples, and the results are shown in Fig 6B. The AUC value of the training set was 0.822, and that of the test set was 0.815. This indicated that the LR model of candidate biomarkers PI (18:0/20:3)-H and PE (18:1p/22:6)-H had a good effect on the sample classification of the test set. When the cutoff of the binary classification model was 0.83, the accuracy of the model in the test set was 0.74, the specificity was 0.88, and the sensitivity was 0.7.

**Table 2:** Logistic regression coefficients of the biomarkers.

Description	Coefficients	Description
(Intercept)	-19.62541206	
NEG1033	2.249933588	PI (18:0/20:3)-H
NEG877	0.634268314	PE (18:1p/22:6)-H

## Discussion

PCOS is a multifactorial disease associated with reproductive and endocrine organs that may lead to infertility and metabolic abnormalities during reproductive age [30]. The application of metabolomics provides a broad perspective for PCOS research. It is a valuable and rapidly expanding tool enabling the discovery of new metabolites. This approach also improves the diagnostic process, making treatment more effective [13]. Machine learning has also received increasing attention in medical applications; in addition to being widely used in image recognition, language processing and data mining, it is also used in various techniques for analyzing and interpreting large amounts of data, which can then be used to create predictive models [31, 32]. This study screened the serum biomarkers PI (18:0/20:3)-H and PE (18:1p/22:6)-H in PCOS patients by untargeted lipidomics-integrated machine learning. Based on the feature selection technology of ensemble learning, a more efficient diagnostic marker panel model is constructed for clinical application.

Machine learning methods provide new techniques for integrating and analyzing various omics data, enabling the discovery of new biomarkers. These biomarkers can potentially contribute to accurate disease prediction, patient stratification, and delivery of precision medicine [33]. Ensemble learning method to select biomarkers by integrating multiple machine learning algorithms of the same or different types to choose those potential target analyses that frequently appear in classification models with high accuracy, thus maximizing the stability of biomarkers [34]. Overall, ensemble learning has superior performance compared to conventional single-dimensional learning. This study uses a feature selection technique based on ensemble learning, which integrates statistical tests and a variety of feature selection algorithms currently used in the mainstream. This technology can select a robust set of diagnostic substances (such as proteins, metabolites, etc.) and build a more efficient diagnostic marker panel model. Analyzing the classification of differential lipid metabolites and the association between differential lipid metabolites and clinical indexes, we demonstrated that different lipid metabolites differ between groups and have close effects on corresponding clinical indexes.

Compared to traditional diagnostic methods, integrated learning methods also have multiple advantages in biomarker discovery in biomedical research, especially in processing complex, high-dimensional biomedical data [35, 36]. For example, biomedical data contains multiple types, such as genome, proteome, metabolome, etc. Integrated learning methods can effectively integrate these multimodal data and reveal complex relationships between different data types. Integrated learning methods usually have stronger generalization capabilities than single models and can reduce prediction errors and improve diagnostic accuracy by combining multiple predictions. Integrated learning methods can improve the speed and efficiency of data processing by processing data in parallel, which is particularly important for biomedical research that requires processing large amounts of data. Integrated learning models are capable of discovering potential biomarkers from large amounts of complex biomedical data that may be difficult to detect in traditional methods. In our study, we screened the key serum biomarkers PI (18:0/20:3)-H and PE (18:1p/22:6)-H through an ensemble learning approach. While the diagnostic panel model had an AUC value of 0.815 in the test set, it had an accuracy of 0.74, a specificity of 0.88, and a sensitivity of 0.7. This indicates that a more efficient diagnostic marker panel model has been constructed.

Biofilms are mainly composed of structural glycerophospholipids, such as phosphatidylinositol (PI) and phosphatidylethanolamine (PE) [37]. PI is the product of different phosphatidylinositol kinases and phosphorylation states at positions D3, D4 and D5 of the inositol ring [38]. It is necessary for many cell signaling pathways of membrane-associated proteins [39]. PE is the major phospholipid of all eukaryotic cells. It can be synthesized by the CDP-ethanolamine branch of the Kennedy pathway, by decarboxylation of phosphatidylserine, or by base exchange with phosphatidylserine [40]. Insulin resistance in PCOS is characterized by abnormal post-receptor signaling in the PI3-kinase pathway [41]. PE plays a vital role in contractile ring disassembly at the cleavage furrow during cytokinesis in mammalian cells [42]. Moreover, PE tends to form a non-lamellar membrane structure and modulates the membrane curvature [43]. Studies have shown that apoptosis of ovarian granulosa cells could be reduced by regulating lipid metabolism to improve ovulation dysfunction in PCOS patients [44]. Qian Y et al. collected follicular fluid and granular cell samples of PCOS women and ordinary women who underwent *in vitro* fertilization and embryo transfer and analyzed lipid metabolomics, showing abnormal metabolism of glycerol, phospholipid and sphingosphayelin in follicular fluid of PCOS women [45]. There are significant lipid differences between normal-weight women with PCOS and normal-weight women without PCOS at the same time, and these different lipid species belong to lipid subclasses, including PE and PI [46]. The PCOS vs luteal phase model showed decreased levels of PE in PCOS women compared with controls [47]. Furthermore, Moran LJ, et al reported that lipidomic signatures were mainly associated with central adiposity and free androgen index in women with and without PCOS. Free androgen index was positively associated with PE and PI [48]. In this paper, our study identifies the critical roles of serum biomarkers PI (18:0/20:3)-H and PE (18:1p/22:6)-H in diagnosing PCOS.

Additionally, different machine learning feature selection approaches could be applied effectively to identify biomarkers. However, each approach had advantages and disadvantages, and biomarkers produced using various feature selection approaches were usually inconsistent due to the multifactorial nature of the features [49]. Therefore, we utilize an ensemble framework, an ensemble learning approach for multi-step data mining that integrates multiple statistical and machine learning methods. This ensemble algorithm identifies and characterizes robust and precise biomarkers with broad applicability and could be applied to build biomarker models on different omics datasets. Simultaneously, biomarkers screened by ensemble machine learning have good classification performance, strong stability and a minor number.

Overall, accurate identification of important biomarkers associated with diseases can provide valuable insights into their underlying etiology and offer great potential for early diagnosis of diseases, personalized medicine, drug response prediction, clinical trials, drug development, and accurate prediction of disease risk. These application prospects indicate that the application of integrated learning in biomedical research has a wide range of potential to improve the efficiency and effectiveness of disease management significantly. With technological advances and improved data analysis methods, future biomarker studies will be more accurate and efficient.

However, in this study, the error metrics (accuracy, sensitivity, and specificity) show a relatively lower model performance compared with other works, such as those by Yu Y et al. [50]. In another work, even only with symptoms and anthropomorphic variables, the Boosting models can achieve a better performance [51]. The performance and variables selected in this study are too few. Variable selection is not a priority either; the two most discriminant features were chosen without considering multiple tests. In the future, to improve the current study, using more variables in the classifier model may potentially improve the performance. As in Table 1, Age LH, FSH, LH/FSH, and T have small P values. Including these and other substance variables in correlation study and PCA might improve the prediction model.

Our study has an additional limitation. The clinical samples collected in this study were randomly collected. Statistically, we found that PCOS patients were younger than non-PCOS subjects. Age might affect the results of this study, but PCOS patients were younger. The age difference might be related to the small sample size, and we will also increase the sample size for further research in future studies.

This study’s dataset needs to be more balanced, with a 3:1 ratio. The unbalanced dataset is known to be demanding for this kind of ML approach. ROC may not be the most appropriate way to evaluate performance, and precision-recall curves are needed as a complement in the future. Furthermore, in a metabolomics study, we should include one or more replication cohorts to validate in the future.

## Conclusions

In this experiment, two biomarkers, PI (18:0/20:3)-H and PE (18:1p/22:6)-H, were finally screened through the analysis of lipidomic data. When the AUC value of the test set of the diagnostic panel model constructed by biomarker was 0.815, the accuracy, specificity, and sensitivity of the model in the test set were 0.74, 0.88, and 0.7, respectively.

## Abbreviations

PCOS: Polycystic ovary syndrome
LR: Logistic regression
RF: Random forest
SVM: Support vector machine
LH: Luteinizing hormone
ESHRE/ASRM: European Society of Human Reproduction and Embryology/American Society for Reproductive Medicine
OPLS-DA: Orthogonal partial least squares discriminant analysis
VIP: Variable Importance for the Projection
ROC: Receiver operating characteristic
AUC: Area under curve
FPR: false positive rate
TPR: actual positive rate
BMI: Body mass index
P: Progesterone
PRL: Prolactin
E2: Estradiol
FSH: Follicle stimulating hormone
T: Testosterone
PI: Phosphatidylinositol
PE: Phosphatidylethanolamine

## References

[1] Gomez JMD, VanHise K, Stachenfeld N, Chan JL, Merz NB, Shufelt C. Subclinical cardiovascular disease and polycystic ovary syndrome. Fertil Steril. 2022;117(5):912–23. doi: 10.1016/j.fertnstert.2022.02.028 35512975 PMC10322116

[2] Ma N, Zhou J, Lu W. The Normal Body Mass Index (BMI) of Women with Polycystic Ovary Syndrome (PCOS) was Associated with IVF/ICSI Assisted Conception Outcomes. Clinical and Experimental Obstetrics & Gynecology. 2023;50(11). doi: 10.31083/j.ceog5011228

[3] Krentowska A, Kowalska I. Metabolic syndrome and its components in different phenotypes of polycystic ovary syndrome. Diabetes Metab Res Rev. 2022;38(1):e3464. doi: 10.1002/dmrr.3464 33988288

[4] Azin F, Khazali H. Phytotherapy of polycystic ovary syndrome: A review. Int J Reprod Biomed. 2022;20(1):13–20. doi: 10.18502/ijrm.v20i1.10404 35308325 PMC8902792

[5] Wright PJ, Corbett CF, Dawson RM, Wirth MD, Pinto BM. Fitness Assessments of Women with Polycystic Ovary Syndrome: A Prospective Process Feasibility Study. Clinical and Experimental Obstetrics & Gynecology. 2023;50(4). doi: 10.31083/j.ceog5004088

[6] Sadeghi HM, Adeli I, Calina D, Docea AO, Mousavi T, Daniali M, et al. Polycystic Ovary Syndrome: A Comprehensive Review of Pathogenesis, Management, and Drug Repurposing. Int J Mol Sci. 2022;23(2). doi: 10.3390/ijms23020583 35054768 PMC8775814

[7] Alesi S, Ghelani D, Mousa A. Metabolomic Biomarkers in Polycystic Ovary Syndrome: A Review of the Evidence. Semin Reprod Med. 2021;39(3–04):102–10. doi: 10.1055/s-0041-1729841 33946122

[8] van der Ham K, Louwers YV, Laven JSE. Cardiometabolic biomarkers in women with polycystic ovary syndrome. Fertil Steril. 2022;117(5):887–96. doi: 10.1016/j.fertnstert.2022.03.008 35512973

[9] Halder A, Kumar H, Sharma M, Jain M, Kalsi AK, Pandey S. Serum anti-Müllerian hormone: A potential biomarker for polycystic ovary syndrome. Indian J Med Res. 2023;158(4):397–406. doi: 10.4103/ijmr.IJMR_4608_20 37991331 PMC10793823

[10] Varikasuvu SR, Pérez-López FR, Ganguly A, Kumar S, Pandi A, Prasad J, et al. Insulin resistance is associated with increased circulating lipocalin-2 levels in polycystic ovary syndrome: a systematic review and meta-analysis. Minerva Endocrinol (Torino). 2023. doi: 10.23736/S2724-6507.22.03926-4 36645405

[11] Zhang CH, Liu XY, Wang J. Essential Role of Granulosa Cell Glucose and Lipid Metabolism on Oocytes and the Potential Metabolic Imbalance in Polycystic Ovary Syndrome. Int J Mol Sci. 2023;24(22). doi: 10.3390/ijms242216247 38003436 PMC10671516

[12] Jové M, Pradas I, Naudí A, Rovira-Llopis S, Bañuls C, Rocha M, et al. Lipidomics reveals altered biosynthetic pathways of glycerophospholipids and cell signaling as biomarkers of the polycystic ovary syndrome. Oncotarget. 2018;9(4):4522–36. doi: 10.18632/oncotarget.23393 29435121 PMC5796992

[13] Rajska A, Buszewska-Forajta M, Rachoń D, Markuszewski MJ. Metabolomic Insight into Polycystic Ovary Syndrome-An Overview. Int J Mol Sci. 2020;21(14). doi: 10.3390/ijms21144853 32659951 PMC7402307

[14] Zhou L, Ni Z, Yu J, Cheng W, Cai Z, Yu C. Correlation Between Fecal Metabolomics and Gut Microbiota in Obesity and Polycystic Ovary Syndrome. Front Endocrinol (Lausanne). 2020;11:628. doi: 10.3389/fendo.2020.00628 33013704 PMC7505924

[15] Omabe M, Elom S, Omabe KN. Emerging Metabolomics Biomarkers of Polycystic Ovarian Syndrome; Targeting the Master Metabolic Disrupters for Diagnosis and Treatment. Endocr Metab Immune Disord Drug Targets. 2018;18(3):221–9. doi: 10.2174/1871530318666180122165415 29359679

[16] Wishart DS. Metabolomics for Investigating Physiological and Pathophysiological Processes. Physiol Rev. 2019;99(4):1819–75. doi: 10.1152/physrev.00035.2018 31434538

[17] Di Minno A, Gelzo M, Stornaiuolo M, Ruoppolo M, Castaldo G. The evolving landscape of untargeted metabolomics. Nutr Metab Cardiovasc Dis. 2021;31(6):1645–52. doi: 10.1016/j.numecd.2021.01.008 33895079

[18] Wang L, Huang S, Zhu T, Ge X, Pei C, Hong G, et al. Metabolomic Study on Iohexol-Induced Nephrotoxicity in Rats Based on NMR and LC-MS Analyses. Chem Res Toxicol. 2022;35(2):244–53. doi: 10.1021/acs.chemrestox.1c00299 35081708

[19] Handelman GS, Kok HK, Chandra RV, Razavi AH, Lee MJ, Asadi H. eDoctor: machine learning and the future of medicine. J Intern Med. 2018;284(6):603–19. doi: 10.1111/joim.12822 30102808

[20] Radakovich N, Nagy M, Nazha A. Machine learning in haematological malignancies. Lancet Haematol. 2020;7(7):e541–e50. doi: 10.1016/S2352-3026(20)30121-6 32589980

[21] Mincholé A, Camps J, Lyon A, Rodríguez B. Machine learning in the electrocardiogram. J Electrocardiol. 2019;57s:S61–s4. doi: 10.1016/j.jelectrocard.2019.08.008 31521378

[22] Silva IS, Ferreira CN, Costa LBX, Sóter MO, Carvalho LML, de CAJ, et al. Polycystic ovary syndrome: clinical and laboratory variables related to new phenotypes using machine-learning models. J Endocrinol Invest. 2022;45(3):497–505. doi: 10.1007/s40618-021-01672-8 34524677

[23] Zhang X, Liang B, Zhang J, Hao X, Xu X, Chang HM, et al. Raman spectroscopy of follicular fluid and plasma with machine-learning algorithms for polycystic ovary syndrome screening. Mol Cell Endocrinol. 2021;523:111139. doi: 10.1016/j.mce.2020.111139 33359305

[24] Revised 2003 consensus on diagnostic criteria and long-term health risks related to polycystic ovary syndrome. Fertil Steril. 2004;81(1):19–25. doi: 10.1016/j.fertnstert.2003.10.004 14711538

[25] Fan S, Kind T, Cajka T, Hazen SL, Tang WHW, Kaddurah-Daouk R, et al. Systematic Error Removal Using Random Forest for Normalizing Large-Scale Untargeted Lipidomics Data. Anal Chem. 2019;91(5):3590–6. doi: 10.1021/acs.analchem.8b05592 30758187 PMC9652764

[26] Taguchi R, Ishikawa M. Precise and global identification of phospholipid molecular species by an Orbitrap mass spectrometer and automated search engine Lipid Search. J Chromatogr A. 2010;1217(25):4229–39. doi: 10.1016/j.chroma.2010.04.034 20452604

[27] Sun Y, Song K, Liu L, Sun L, Qin Q, Jiang T, et al. Sulfoquinovosyl diacylglycerol synthase 1 impairs glycolipid accumulation and photosynthesis in phosphate-deprived rice. J Exp Bot. 2021;72(18):6510–23. doi: 10.1093/jxb/erab300 34165534

[28] Yang L, Xuan C, Yu C, Zheng P, Yan J. Diagnostic Model of Alzheimer’s Disease in the Elderly Based on Protein and Metabolic Biomarkers. J Alzheimers Dis. 2022;85(3):1163–74. doi: 10.3233/JAD-215119 34924381

[29] Schisterman EF, Perkins NJ, Liu A, Bondell H. Optimal cut-point and its corresponding Youden Index to discriminate individuals using pooled blood samples. Epidemiology. 2005;16(1):73–81. doi: 10.1097/01.ede.0000147512.81966.ba 15613948

[30] Heidarzadehpilehrood R, Pirhoushiaran M, Abdollahzadeh R, Binti Osman M, Sakinah M, Nordin N, et al. A Review on CYP11A1, CYP17A1, and CYP19A1 Polymorphism Studies: Candidate Susceptibility Genes for Polycystic Ovary Syndrome (PCOS) and Infertility. Genes (Basel). 2022;13(2). doi: 10.3390/genes13020302 35205347 PMC8871850

[31] Abbasi B, Goldenholz DM. Machine learning applications in epilepsy. Epilepsia. 2019;60(10):2037–47. doi: 10.1111/epi.16333 31478577 PMC9897263

[32] Clarke SL, Parmesar K, Saleem MA, Ramanan AV. Future of machine learning in paediatrics. Arch Dis Child. 2022;107(3):223–8. doi: 10.1136/archdischild-2020-321023 34301619

[33] Reel PS, Reel S, Pearson E, Trucco E, Jefferson E. Using machine learning approaches for multi-omics data analysis: A review. Biotechnol Adv. 2021;49:107739. doi: 10.1016/j.biotechadv.2021.107739 33794304

[34] Baggerly KA, Morris JS, Edmonson SR, Coombes KR. Signal in noise: evaluating reported reproducibility of serum proteomic tests for ovarian cancer. J Natl Cancer Inst. 2005;97(4):307–9. doi: 10.1093/jnci/dji008 15713966

[35] Guo R, Teng Z, Wang Y, Zhou X, Xu H, Liu D. Integrated Learning: Screening Optimal Biomarkers for Identifying Preeclampsia in Placental mRNA Samples. Comput Math Methods Med. 2021;2021:6691096. doi: 10.1155/2021/6691096 33680070 PMC7925050

[36] Xie S, Zeng D, Wang Y. Integrative network learning for multi-modality biomarker data. Ann Appl Stat. 2021;15(1):64–87. doi: 10.1214/20-aoas1382 34354791 PMC8329868

[37] Hirano T, Sato MH. Diverse Physiological Functions of FAB1 and Phosphatidylinositol 3,5-Bisphosphate in Plants. Front Plant Sci. 2019;10:274. doi: 10.3389/fpls.2019.00274 30967882 PMC6439278

[38] Desale SE, Chinnathambi S. Phosphoinositides signaling modulates microglial actin remodeling and phagocytosis in Alzheimer’s disease. Cell Commun Signal. 2021;19(1):28. doi: 10.1186/s12964-021-00715-0 33627135 PMC7905611

[39] Kimble-Hill AC, Petrache HI, Seifert S, Firestone MA. Reorganization of Ternary Lipid Mixtures of Nonphosphorylated Phosphatidylinositol Interacting with Angiomotin. J Phys Chem B. 2018;122(35):8404–15. doi: 10.1021/acs.jpcb.7b12641 29877706 PMC6351316

[40] Signorell A, Rauch M, Jelk J, Ferguson MA, Bütikofer P. Phosphatidylethanolamine in Trypanosoma brucei is organized in two separate pools and is synthesized exclusively by the Kennedy pathway. J Biol Chem. 2008;283(35):23636–44. doi: 10.1074/jbc.M803600200 18587155 PMC3259767

[41] Barber TM, Franks S. Obesity and polycystic ovary syndrome. Clin Endocrinol (Oxf). 2021;95(4):531–41. doi: 10.1111/cen.14421 33460482

[42] Martin SJ, Reutelingsperger CP, McGahon AJ, Rader JA, van Schie RC, LaFace DM, et al. Early redistribution of plasma membrane phosphatidylserine is a general feature of apoptosis regardless of the initiating stimulus: inhibition by overexpression of Bcl-2 and Abl. J Exp Med. 1995;182(5):1545–56. doi: 10.1084/jem.182.5.1545 7595224 PMC2192182

[43] Lucki NC, Li D, Bandyopadhyay S, Wang E, Merrill AH, Sewer MB. Acid ceramidase (ASAH1) represses steroidogenic factor 1-dependent gene transcription in H295R human adrenocortical cells by binding to the receptor. Mol Cell Biol. 2012;32(21):4419–31. doi: 10.1128/MCB.00378-12 22927646 PMC3486137

[44] Zhu H, Wu Y, Zhuang Z, Xu J, Chen F, Wang Q, et al. Ampelopsis japonica aqueous extract improves ovulatory dysfunction in PCOS by modulating lipid metabolism. Biomed Pharmacother. 2024;170:116093. doi: 10.1016/j.biopha.2023.116093 38159378

[45] Qian Y, Tong Y, Zeng Y, Huang J, Liu K, Xie Y, et al. Integrated lipid metabolomics and proteomics analysis reveal the pathogenesis of polycystic ovary syndrome. J Transl Med. 2024;22(1):364. doi: 10.1186/s12967-024-05167-x 38632610 PMC11022415

[46] Ban Y, Ran H, Chen Y, Ma L. Lipidomics analysis of human follicular fluid form normal-weight patients with polycystic ovary syndrome: a pilot study. J Ovarian Res. 2021;14(1):135. doi: 10.1186/s13048-021-00885-y 34645507 PMC8515674

[47] Haoula Z, Ravipati S, Stekel DJ, Ortori CA, Hodgman C, Daykin C, et al. Lipidomic analysis of plasma samples from women with polycystic ovary syndrome. Metabolomics. 2015;11(3):657–66. doi: 10.1007/s11306-014-0726-y 25972770 PMC4419155

[48] Moran LJ, Mundra PA, Teede HJ, Meikle PJ. The association of the lipidomic profile with features of polycystic ovary syndrome. J Mol Endocrinol. 2017;59(1):93–104. doi: 10.1530/JME-17-0023 28500248

[49] Baggerly KA, Morris JS, Coombes KR. Reproducibility of SELDI-TOF protein patterns in serum: comparing datasets from different experiments. Bioinformatics. 2004;20(5):777–85. doi: 10.1093/bioinformatics/btg484 14751995

[50] Yu Y, Tan P, Zhuang Z, Wang Z, Zhu L, Qiu R, et al. Untargeted metabolomic approach to study the serum metabolites in women with polycystic ovary syndrome. BMC Med Genomics. 2021;14(1):206. doi: 10.1186/s12920-021-01058-y 34416878 PMC8379735

[51] Zigarelli A, Jia Z, Lee H. Machine-Aided Self-diagnostic Prediction Models for Polycystic Ovary Syndrome: Observational Study. JMIR Form Res. 2022;6(3):e29967. doi: 10.2196/29967 35289757 PMC8965679

